# Minimally invasive colostomy with endoscopy as a novel technique for creation of a trephine stoma

**DOI:** 10.1038/s41598-021-96357-w

**Published:** 2021-08-17

**Authors:** Teppei Kamada, Hironori Ohdaira, Junji Takahashi, Wataru Kai, Keigo Nakashima, Yuichi Nakaseko, Norihiko Suzuki, Masashi Yoshida, Yutaka Suzuki

**Affiliations:** Department of Surgery, International University of Health and Welfare Hospital, 537-3, Iguchi, Nasushiobara, Tochigi 329-2763 Japan

**Keywords:** Colonoscopy, Gastrointestinal diseases, Colorectal cancer

## Abstract

The conventional approach of trephine stoma creation is associated with various limitations, including poor elevation of the sigmoid colon, misidentification of the target organs, and poor visualization of the operative field, which may require conversion to an open approach. Our study aimed to evaluate the safety, feasibility, and complications of minimally invasive colostomy with endoscopy (MICE), a new technique for trephine stoma creation. This retrospective cohort study included 14 patients. Patients diagnosed with obstructive rectal cancer or bladder and rectal disorders due to spinal cord injury or bone metastasis requiring sigmoid loop colostomy were eligible for the procedure. MICE was performed using a combination of endoscopic and fluoroscopic procedures. The primary endpoint was the technical success of MICE. Technical success using MICE was achieved in all 14 cases. The mean total operative time was 52.6 (range 32–107) min, and mean blood loss was 18.9 (range 1–50) mL. There was no incidence of conversion to open surgery. Postoperative complications included peristomal abscess formation and ischemic colitis in each case. MICE may be useful as a minimally invasive approach for trephine stoma creation that overcomes the problems of a conventional approach in high-risk patients.

## Introduction

Colostomy creation is an essential procedure to manage defecation function in patients with obstructive colorectal cancer or spinal cord injury^1–3^. As these health conditions also negatively impact physical status, a minimally invasive technique to manage defecation would be desirable. The method of colostomy creation, by laparotomy or laparoscopy, is generally selected based on a surgeon’s preference. However, conventional open methods are associated with postoperative pain and complications that can include ileus and wound infection; these complications can delay the introduction of postoperative treatment, such as chemotherapy^4^. A trephine stoma is a less invasive technique for colostomy creation than an open approach^5^ and, thus, can be a useful option for patients with health comorbidities, as well as preventing postoperative complications related to an open surgical wound. However, the creation of a trephine stoma is technically difficult and is itself associated with a complication rate of about 20%, including poor elevation of the sigmoid colon and misidentification of the target organs, as well as a poor visualization of the surgical field^6,7^, which can require conversion to an open approach.

Since 2015, we have been accumulating experiences performing a minimally invasive colostomy with endoscopy (MICE) approach that preserves the advantages of combining conventional trephine stoma approach with endoscopic and fluoroscopic procedures. Therefore, the study aimed to evaluate the safety, feasibility, and complications of MICE for trephine stoma creation at our hospital.

## Methods

### Statement of Ethics

Informed consent was obtained from all patients. The MICE procedure was approved by the Institutional Review Board of the International University of Health and Welfare Hospital (Approval No. 13-B-97). This study was performed in accordance with relevant guidelines/regulations, and informed consent was obtained from all participants or their guardians. This study was performed in accordance with the Declaration of Helsinki.

### Study design and inclusion/exclusion criteria

This was a retrospective cohort study of patients who were eligible for the MICE procedure at a single center, the International University of Health and Welfare Hospital (Nasushiobara, Tochigi prefecture, Japan), between November 2015 and November 2020. The inclusion criteria were high-risk patients with American Society of Anesthesiologists (ASA) physical status classification scores of ≥ 3 diagnosed with obstructive rectal cancer or bladder and rectal disorders due to spinal cord injury or bone metastasis who required a sigmoid loop colostomy for defecation control. Patients who were unable to undergo endoscopy due to severe stenosis and those in whom informed consent for MICE could not be obtained were excluded.

### Endpoints of the study

The primary endpoint of our study was the technical success of MICE. The secondary endpoints were clinical success (achieving intestinal decompression or good defecation control), operative time, blood loss, intra- and postoperative complications, and delay to the start of oral intake after surgery.

### Preoperative evaluation

All patients were evaluated for surgical tolerance under general or spinal anesthesia by preoperative examinations, including electrocardiogram, echocardiography, spirogram, and blood test.

Preoperative colonoscopy was performed to confirm whether the colonoscope could pass through the obstructed lesion. In addition, preoperative computed tomography was performed to confirm that the sigmoid colon had sufficient length for stoma creation and the absence of other organs, such as the small intestine, intervening between the abdominal wall and the sigmoid-descending colon (SD) junction in the axial plane. The sufficient length for stoma creation was defined as a flexible sigmoid colon with one or more loops.

### Surgical procedure

The day prior to surgery, mechanical bowel preparation was performed using magnesium citrate for patients without obstructive symptoms. Insertion of transanal ileus tube and transanal irrigation were performed to gain a clear endoscopic view for patients with obstructive symptoms. As well, the location for the stoma was marked in two places on the left abdominal wall by a stoma therapist. The MICE trephine stoma procedure was performed by a team of two surgeons, under constant fluoroscopic guidance, with the patient in the lithotomy position, under general or spinal anesthesia. Antibiotic prophylaxis was provided in all cases: cefmetazole (1 g), administered 30 min before incision, and an additional dose administered every 3 h during the surgery. Carbon dioxide was used for endoscopic insufflation.

As a first step, a colonoscopy was performed to reach the SD junction. As previously stated, if the colonoscope could not be passed through the obstructive lesion, a gastroscope or nasal endoscope was used. Then, the mobility of the sigmoid colon was confirmed by performing a push–pull maneuver of the endoscope around the anus and the SD junction under fluoroscopy. If the sigmoid colon was poorly mobile, MICE was judged to be impossible, and the procedure was converted to conventional laparoscopy or laparotomy for colostomy creation. If the sigmoid was judged to be sufficiently mobile, we proceeded with MICE.

**Figure 1:**
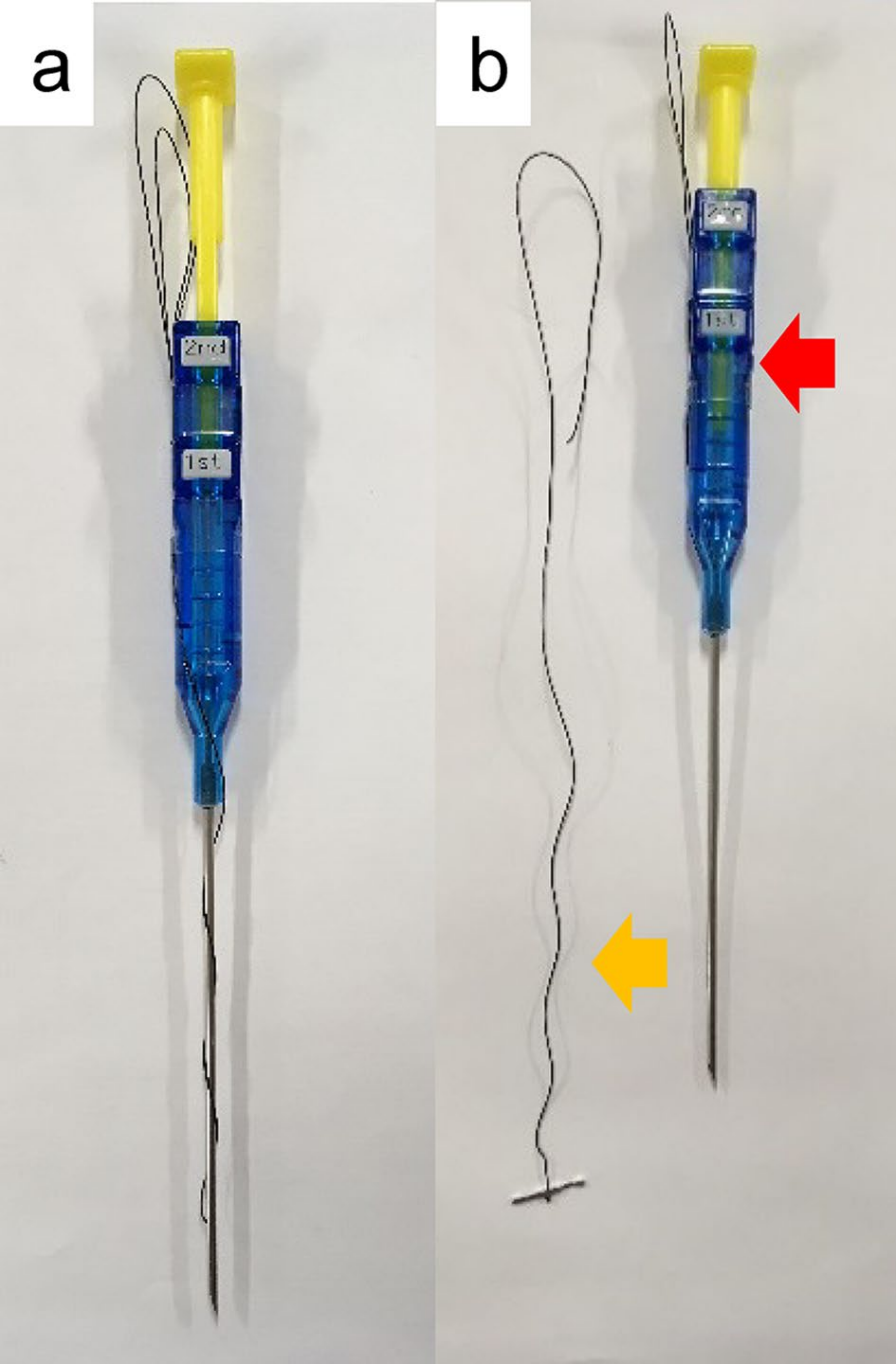
Two-shot anchor device containing a thread with metal bar attached (T-bar), containing two threads within one guiding needle, each thread having a metal bar. (**a**) Two shot anchors before puncturing and (**b**) button (red arrow), which is pushed to separate the thread with the T-bar from the tip of the device (yellow arrow).

**Figure 2:**
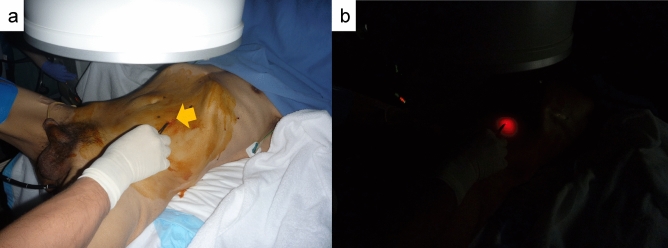
Identification of the site for puncture using (**a**) the finger pressing test (which involves pressing down the abdominal wall with a finger or Pean forceps under fluoroscopy) and (**b**) the illumination test to confirm that the sigmoid-descending colon (SD) junction is directly below the target site of puncture.

**Figure 3:**
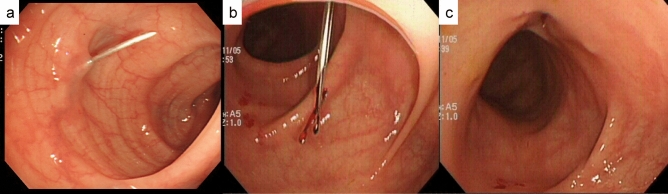
Puncture of the sigmoid colon and placement of the thread with a metal bar attached (T-bar). (**a**) An exploratory puncture is made using a 23-gauge needle. (**b**,**c**) The 2-shot anchor device is inserted into the interior space of the sigmoid colon, and the thread with T-bar is detached.

**Figure 4:**
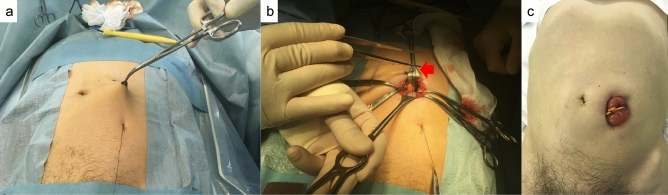
Creation of the colostomy. (**a**) The nylon thread is pulled out of the body, and a circular skin incision is performed at the site previously marked for stoma position. (**b**) The nylon thread in the abdominal cavity is identified through the site of puncture (red arrow). (**c**) The sigmoid loop colostomy is created without incision.

To identify the sigmoid colon, a 2-shot anchor device (Olympus, Tokyo, Japan) was used (Fig. 1a,b). This anchor device was originally designed to anchor the stomach to the abdominal wall during percutaneous endoscopic gastrostomy. A ‘finger press test’ and ‘illumination test’ were performed to confirm the SD junction was directly below the target site for puncture (Fig. 2a,b). An exploratory puncture was performed, using a 23-gauge needle for further confirmation of the site for puncture and fixation (Fig. 3a). A suction test was used to confirm the absence of intervening organs between the sigmoid colon and the abdominal wall. A cutaneous incision, approximately 2 mm in length, was created at the target site for puncture using a scalpel; the incisional space was then enlarged using Pean forceps. The 2-shot anchor device was inserted into the interior space of the sigmoid colon, and the thread with a metal T-bar was detached and pulled toward the surface of the body (Fig. 3b,c). If the SD junction could not be punctured, another site in the sigmoid colon was punctured using the same procedure. One or two stitches were used for this puncture procedure as required. The nylon thread was then pulled through the skin incision. A circular incision was then performed at the stoma site previously marked on the left abdominal wall, through the skin and subcutaneous tissue to reach the anterior layer of the rectus sheath (Fig. 4a). The anterior layer of the rectus sheath was incised in a cruciate fashion, and the rectus abdominis muscle split along its fibers. The posterior layer of the rectus sheath was then incised, and the peritoneum opened. The nylon threads that had been placed at the puncture site from outside the body were identified within the abdominal cavity, and the oral and anal sides of the sigmoid colon were identified by illumination of the sigmoidoscopy. The sigmoid colon was then pulled out of the body through the trephine hole (Fig. 4b). At this point, if it was difficult to raise the sigmoid colon to the outside of the body due to adhesion to the surrounding organs or lateral peritoneum, the sigmoid colon was suspended from the abdominal wall using the nylon thread and adhesions peeled from the trephine hole to allow the sigmoid colon to be sufficiently raised. A small hole was created in the mesentery and raised using a flexible catheter to prevent the sigmoid colon from returning into the abdominal cavity (Fig. 4c). Finally, the intestinal wall and subcutaneous tissue were fixed using a 12–16 point, 3-0 vicryl suture such that the mucosa was inverted and the sigmoid loop colostomy was created (Fig. 4c).

## Results

### Characteristics of the study patients

The characteristics of the study population are reported in Table 1, with relevant features summarized as follows. Our study population included 14 patients, including nine men, with a mean age of 70 (range 47–90) years. Of the 14 patients included, eight had an ASA physical status classification scores of 3, and six had a score of 4. The primary disease was rectal cancer in nine cases, esophageal cancer in two, breast cancer in two, and spinal cord injury in one. The indication for stoma creation was the prevention of bowel obstruction in nine cases and bladder and rectal disorders in five cases. The stoma procedure was performed under general anesthesia in 13 cases and spinal anesthesia in 1 case. Six patients had a previous history of abdominal surgery. For the nine patients with rectal cancer, two required additional radical resections after MICE, and four received postoperative chemotherapy; the other three did not receive additional postoperative treatment. The mean serum albumin level was 3.3 (range 2.4–4.2) g/dL, and the mean body mass index (BMI) was 19.4 (range 15.8–23.2) kg/m^2^. The mean Onodera’s Prognostic Nutrition Index (PNI)^8^ was 37.9 (range 27.3–49.4).

**Table 1:** Patient characteristics and procedures.

Case	Age Sex	Primary disease	Surgical history	Stoma indication	ASA-PS	Puncture site	Adhesiolysis	Operative time (min)	Puncture number	Blood loss (mL)	Complication	Duration to chemotherapy (days)	Follow-up (months)
1	80 W	Rectal cancer	Appendectomy	Obstruction	3	S	−	54	2	10	−	7	5
2	69 M	Rectal cancer	–	Obstruction	3	SDJ	+	66	2	5	Ischemic colitis	14	6
3	77 M	Rectal cancer	–	Obstruction	4	S	−	52	2	50	Abscess around stoma	−	25
4	80 W	Rectal cancer	–	Obstruction	3	S	−	43	1	10	−	−	9
5	65 M	Rectal cancer	–	Obstruction	3	S	−	45	2	50	−	3	14
6	69 W	Breast cancer	Appendectomy	Defecation Dysfunction	3	SDJ	−	43	2	3	−	−	2
7	65 M	Rectal cancer	–	Obstruction	4	S	−	35	2	5	−	7	2
8	90 W	Rectal cancer	Bowel resection	Obstruction	3	S	−	52	2	50	−	−	35
9	52 M	Spinal cord injury	Bladder puncture	Defecation Dysfunction	3	S	−	42	2	1	−	−	1
10	47 W	Breast cancer	–	Defecation Dysfunction	4	SDJ	−	59	2	10	−	−	5
11	74 M	Esophageal cancer	Esophagectomy	Defecation Dysfunction	4	S	−	48	1	10	−	−	4
12	79 M	Rectal cancer	–	Obstruction	4	SDJ	+	107	1	50	−	−	4
13	64 M	Rectal cancer	–	Obstruction	4	S	−	59	1	5	−	7	9
14	68 M	Esophageal cancer	Esophagectomy	Defecation Dysfunction	3	S	−	32	1	5	−	−	4

ASA-*PS* American Society of Anesthesiologists physical status classification, *S* sigmoid colon, *SDJ* sigmoid-descending colon junction.

### Surgical outcome

The surgical outcomes are reported in Table 1. Technical success of the MICE procedure was achieved in all cases. Clinical success was achieved in all cases. We note that good mobility of the sigmoid colon was confirmed on preoperative fluoroscopic colonoscopy examination in all cases. The mean operative time was 52.6 (range 32–107) min, which included a mean endoscopic fixation time of 9.4 (range 3–20) min and a mean colostomy creation time of 43.2 (range 29–92) min. The mean volume of blood loss was 18.9 (range 1–50) mL. In nine cases, two fixations (using the 2-shot anchor device) were required, with one fixation required in the other five cases. The puncture site was at the SD junction in four cases and the sigmoid colon in 10. The delay to the initiation of eating was 1 day in 13 cases and 2 days in one case. Adhesion peeling was immediately under the trephine hole due to poor intestinal elevation because of intra-abdominal adhesion, which was required in two cases. One of these cases required that the trephine hole be extended by 1 cm in the cranial and caudal direction. Postoperative complications included peristomal abscess formation in one case and ischemic colitis in one case. These patients improved with conservative treatment with antibiotics. For the five patients requiring postoperative chemotherapy, the mean duration to treatment initiation after surgery was 7.6 (range 3–14) days. The mean follow-up period was 8.9 (range 1–35) months. Of the 14 patients included in our study, seven (50%) died due to the worsening of their underlying disease.

## Discussion

The greatest merit of MICE is that it can safely and easily identify the sigmoid colon and guide the sigmoid colon out of the body through the trephine hole without the need for a conventional laparotomy procedure. In this study, MICE was successfully performed in all 14 cases, with no need for conversion to laparotomy.

Conventionally, colostomy creation is performed by either laparotomy or laparoscopy^9–11^. Ivatury et al.^10^ reported that in the American College of Surgeons National Surgical Quality Improvement Program database, colostomy creation was performed more often using an open surgical than laparoscopic approach (2179 and 1132 cases, respectively), with the operative time being significantly shorter for open surgery (81 versus 86 min, respectively). However, the rate of mortality and complication on postoperative day 30 was significantly higher for an open than for a laparoscopic approach: mortality, 8.7% versus 3.5%, respectively, and complication, 25.4% versus 17.0%, respectively. For colostomy creation, a less invasive procedure is desirable but can be technically difficult to perform. However, all conventional approaches require an open wound which can be associated with postoperative complications, such as pain, adhesion formation, wound infection, and abdominal wall dissemination^4^.

The trephine stoma procedure, first described in 1991^5^, uses a minimally invasive approach without the need for laparotomy. Moreover, as there is no surgical wound, there is less postoperative pain and a lower risk of postoperative complications, which can favor rapid introduction of postoperative therapy, such as chemotherapy.

The advantages of the trephine stoma procedure over an open surgical approach have previously been described. In a group of 263 patients who underwent transverse colon stoma creation, Yeom et al.^12^ compared outcomes between trephine (n = 161), open (n = 82), and laparoscopic (n = 20) procedures. The trephine procedure was associated with a shorter operative time (46.0 ± 1.9 min, 78.7 ± 3.9 min, and 63.5 ± 5.0 min, respectively, p < 0.001) and shorter time to flatus (1.8 ± 0.1 days, 2.1 ± 0.1 days, and 2.2 ± 0.3 days, respectively, p = 0.025), but with no difference in the length of hospital stay and rate of postoperative complications among the three procedures. Based on these results, Yeom et al. concluded that the trephine stoma is a safe, useful, and feasible procedure.

However, the trephine stoma procedure tends to be unpopular due to its technical difficulty, including poor elevation of the intestinal tract due to adhesion formation or the length of the intestinal tract, misidentification of the target organs, and poor visualization of the operative field. Patel et al.^13^ reported that among 31 patients who underwent a trephine stoma procedure, nine (29%) required conversion to laparotomy due to difficulty in mobilization of the colon secondary to adhesions. Moreover, the procedure was successful in only four of the seven patients (57%) with a history of prior laparotomy. Overall, to summarize the previous studies, the rate of conversion to an open approach during the trephine stoma approach has been reported to be between 4.3 and 29.0%^5,12–15^.

MICE has the potential to overcome problems associated with the conventional trephine stoma procedure. First, endoscopic-assisted puncture of the sigmoid colon using a 2-shot anchor device ensures that the sigmoid colon is clearly identified. In all our cases, the nylon thread of the T-bar could be easily identified in the abdominal cavity, requiring a mean endoscopic fixation time of only 9.8 min. Intestinal traction to the outside of the body using the nylon thread is also safer, performed under a better field of view than conventional methods to lift the sigmoid colon, including the use of Babcock’s forceps, manual lifting, or using the endoscope through the trephine hole^16–18^. A second advantage is that the indication for MICE can be appropriately evaluated before surgery by confirming the mobility of the sigmoid colon under fluoroscopic guidance. We note that previous studies have not reported on the preoperative assessment of indications for a trephine stoma. Moreover, MICE can be performed technically under local anesthesia; nevertheless, we performed MICE standardly under general anesthesia so that we can switch to the conventional procedure if preoperative evaluation identifies that mobility of the sigmoid colon is poor.

We consider that the high rate of technical success of MICE was related to effective evaluation of endoscopic mobility preoperatively and that the mean BMI of patients included in our study group was low, at a mean of 19.4 kg/m^2^. For cases with a short sigmoid colon and severe adhesion, further adhesion peeling is possible by extending the skin/fasciotomy of the trephine hole in the cranial and caudal directions by about 1 cm. Of clinical significance, in patients with rectal cancer, the MICE trephine stoma procedure provides the added advantage that chemotherapy can be rapidly introduced after surgery without having to wait for wound healing. Arhi et al.^19^ reported that delayed introduction of adjuvant chemotherapy was associated with worse overall survival (hazard ratio [HR], 1.44; 95% confidence interval 1.16–1.79; p = 0.001), with reoperation and wound infection being the main reasons for the delayed introduction of chemotherapy. Among patients with rectal cancer in our case series, the mean delay from surgery to chemotherapy initiation was 7.6 (range 3–14) days. Moreover, rapid introduction of chemotherapy was not associated with any chemotherapy-related complications.

One of the complications of MICE is injury to other organs during sigmoid colon puncture. When the nylon thread was detected outside the abdominal cavity in one case in our case series, the T-bar could not be palpated in the elevated intestinal tract. In this case, the nylon thread was returned into the abdominal cavity; the position of the T-bar was confirmed to be in the sigmoid colon, but the nylon thread had penetrated the transverse colon because of its excessive length. The nylon thread penetrating the transverse colon was removed, and the sigmoid colon was pulled out of the body, with MICE being successfully performed thereafter.

The postoperative course was good with no complications. We believe that the small puncture hole in the colon, performed using the 17-gauge needle of the 2-shot anchor device, was favorable in this regard. In addition, based on our prior experience, we performed a suction test at the time of exploratory puncture, using a 23-gauge needle to confirm that air in the intervening intestinal tract could not be aspirated. The suction test makes it possible to puncture the sigmoid colon reliably, avoiding accidental puncture of other organs. Of note, with a longer duration of the endoscopic procedure, air will enter the small intestine and transverse colon with the resulting dilation causing a poor visual field. Therefore, the endoscopic time should be kept to a minimum, and carbon dioxide should be used.

The limitations of our case series need to be acknowledged. Foremost, this was a single-center retrospective study with a small sample size, and almost all procedures were performed under general anesthesia; therefore, future studies with a larger sample size are warranted to verify results. MICE might be difficult to perform in patients with a short sigmoid colon or the interposition of other organs between the abdominal wall and the colon. Further investigation will be required to determine whether transverse colostomy or end colostomy can be performed using the MICE procedure or MICE can be performed under local anesthesia.

In conclusion, using a detailed preoperative adaptive evaluation, MICE might be useful as a minimally invasive option for trephine stoma creation in patients at high risk for complications and those for whom early initiation of postoperative chemotherapy is indicated, overcoming the problems associated with conventional open trephine stoma creation.

## Data Availability

The data that support the findings of this study are available from the corresponding author upon reasonable request.
